# Methodological Issues on Planning and Running the Brazilian Multicenter Study on Preterm Birth

**DOI:** 10.1155/2015/719104

**Published:** 2015-02-10

**Authors:** Giuliane J. Lajos, Ricardo P. Tedesco, Renato Passini, Tabata Z. Dias, Marcelo L. Nomura, Patrícia M. Rehder, Samira M. Haddad, Maria H. Sousa, Jose G. Cecatti, Brazilian Multicenter Study on Preterm Birth Study Group

**Affiliations:** ^1^Department of Obstetrics and Gynecology, School of Medical Sciences, University of Campinas, 13083-881 Campinas, SP, Brazil; ^2^Center for Studies in Reproductive Health of Campinas (CEMICAMP), 13083-881 Campinas, SP, Brazil; ^3^University of Campinas, 13083-881 Campinas, SP, Brazil

## Abstract

*Objectives*. Assuming that the occurrence of preterm births and their maternal and neonatal associated conditions in Brazil are not completely known, a multicenter study was proposed. The purpose of this paper is to describe the methods used, its processes, achievements, and challenges.* Study Design*. A multicenter cross-sectional study on preterm births in Brazilian facilities plus a nested case-control study to assess their associated factors. A description of all steps of planning and implementing such a nationwide study, including strategies for dealing with problems arising during the process, is presented.* Results*. 20 referral hospitals in different regions of Brazil participated in the study. A detailed questionnaire for data collection, an electronic platform for data transcription and monitoring, research materials, and specific monitoring tools were developed; then data management and analyses were performed. Finally, we got information on 4,150 preterm births and 1,146 term births.* Conclusions*. This study represented the first step of a planned comprehensive assessment of preterm birth in Brazil, with detailed information that will lead to several analyses and further studies, bringing the knowledge to improve screening, diagnosis, and treatment practices in maternal and perinatal health with the final purpose of reducing the burden of this condition in the country.

## 1. Introduction

Preterm birth currently is a major cause of neonatal morbidity and mortality worldwide. Classically defined as the birth that occurs before the 37th week of pregnancy [1], prematurity is the leading cause of newborn deaths and now the second leading cause of death after pneumonia in children under the age of 5 [2]. It is estimated that 15 million babies are born too soon every year and over 1 million children die each year due to complications of preterm birth [3]. Many survivors face a lifetime of disability, including learning disabilities and vision and hearing problems [4].

Preterm birth is a syndrome with a variety of causes, which can be classified in* spontaneous* preterm birth (spontaneous onset of labor or following prelabor premature rupture of membranes, pPROM) and* provider-initiated *preterm birth (defined as induction of labor or elective caesarean birth before 37 completed weeks of gestation for maternal or fetal indications, both “urgent” and “discretionary,” or other nonmedical reasons) [5]. Preterm births are spontaneous in approximately 75% of the cases [6] and its etiology is probably a multifactorial process, resulting from the interplay of factors causing the uterus to change from quiescence to active contractions and to birth before term. The precursors to spontaneous preterm birth vary by gestational age [7] and social and environmental factors, but the cause of spontaneous preterm labor remains unidentified in up to half of all cases [8]. Maternal history of preterm birth is a strong risk factor and most likely is driven by the interaction of genetic, epigenetic, and environmental risk factors [9]. Many other maternal factors have been associated with an increased risk of spontaneous preterm birth, including young or advanced maternal age, short interpregnancy intervals, and low maternal body mass index [10, 11]. Some lifestyle factors that contribute to spontaneous preterm birth include stress and excessive physical work or long times spent standing and smoking and excessive alcohol consumption [11, 12]. Another important risk factor is uterine over distension with multiple pregnancies (twins, triplets, etc.) that carry nearly 10 times higher the risk of preterm birth compared to singleton births [13].

A relevant aspect is the presence of fetal and maternal infections. There is an association between positive cervical cultures and maternal and fetal infectious morbidities, such as urinary tract infections with preterm labor [14]. In addition, other conditions have more recently been shown to be associated with both preterm birth and infection, as the case of cervical insufficiency resulting from ascending intrauterine infection and inflammation with secondary premature cervical shortening [15], and periodontal disease [12, 16]. From those preterm births resulting from medical indication, more than half are associated with preeclampsia, fetal distress, intrauterine fetal growth restriction,* abruptio placentae*, and placental insufficiency [17].

Preterm birth rates are increasing in almost all countries with reliable data [3]. In the United States, for instance, nearly 12 out of every 100 babies born in 2010 were premature, and this rate has increased by 30% since 1981 [18]. This fact has greatly motivated the interest from authorities and those responsible for the different sectors of maternal and child health, either public or private, in several countries of the world.

The official prevalence of preterm birth in Brazil in 2006 was around 6.5%. However, certainly this figure is no longer real. In fact, population-based studies demonstrate that it is higher [19, 20]. The undercount estimate from governmental agencies may be a consequence of difficulties to accurately estimate gestational age, currently adopted information systems that may result in poor records, therefore decreasing their reliability, and significant population differences in a continental-sized country. Late or sometimes nonexistent prenatal care makes it difficult or even impossible to provide a reliable estimate of the gestational age. The same is true regarding the lack of neonatal care during labor, which also contributes to an imprecise estimate of gestational age and, consequently, of the occurrence of preterm birth in country. In addition to easy access to prenatal care, it is imperative to develop a national standard to assess gestational age at birth through the evaluation of the newborn infant, which is essential to implement guidelines in different clinical situations of the obstetrics and pediatrics practices.

Investment in women's and maternal health and care at birth will probably reduce stillbirth rates and improve outcomes for women and newborn babies, especially those who are preterm. Global progress in child survival and health to 2015 and beyond cannot be achieved without addressing preterm birth [21].

Therefore it is possible to conclude that it is important to assess the situation of preterm birth in Brazil (as it would be as well for any other low and middle income country contributing to a large proportion of the burden of preterm birth in the world), knowing its real prevalence and associated socioeconomic factors, currently adopted preventive measures, diagnostic and screening methods applied, interventions, and short and long term maternal and neonatal outcomes. This evidence, in association with that from high-income countries, will guide health professionals and policy makers in applying the necessary preventive and appropriate measures to face this problem.

The purpose of this paper is to describe methodological issues and procedures adopted for building and implementing of the Brazilian Multicenter Study on Preterm Birth (EMIP), which is part of the Brazilian Network for Studies on Reproductive and Perinatal Health and evaluated the prevalence of preterm births in several hospitals in Brazil, and in addition determining their main causal factors, associated risk factors, treatment protocols, and associated perinatal morbidity and mortality, besides its processes, achievements, and challenges, including strategies for dealing with problems arising during the process. This is expected to be helpful, especially for people from low and middle-income countries, for those willing to settle such a kind of research collaboration with similar purposes.

## 2. Materials and Methods

### 2.1. Organization of the Study

This was a multicenter cross-sectional study plus a nested case-control study implemented in referral obstetrical units in different geographical regions of Brazil, under the coordination of the Department of Obstetrics and Gynecology of the School of Medical Sciences, University of Campinas, Brazil. The full research proposal has already been published elsewhere [22]. For the cross-sectional component, the participating centers performed a prospective surveillance of all patients admitted to give birth, in order to identify preterm birth cases and their main causes. In the first months of the study an analysis of the factors associated with spontaneous preterm birth was also carried out, comparing women who had preterm birth with a sample of those who delivered at term. For the whole study, around 37,000 births should be followed, corresponding to approximately half the deliveries of all participating centers in a 12-month period. For the case-control study component, the estimated sample size was 1,055 women in each group (cases and controls). The total number of preterm births estimated to be followed for both components of the study was around 3,600, but finally we got information on 4,150 preterm births and 1,146 term births.

Data was collected through a questionnaire by three ways: interviewing women in the first to third day postpartum, obtaining information in their medical records and prenatal chart, and from the newborn medical records. Then data was then entered in an electronic form and sent electronically to a central database at the coordinating center. Data analysis was performed by subgroups according to gestational age, main determining causes, therapeutic management, and neonatal outcomes. Then, the respective rates, ratios, and relative risks were estimated for the possible predictors.

### 2.2. Selection of the Centers to Constitute the Network

During the National Congress of Gynecology and Obstetrics which occurred in Fortaleza in November 2007, a national network called “Brazilian Network for Studies on Reproductive and Perinatal Health (BNSRPH)” was established. It involved 27 healthcare institutions from around the country, representing the five regions of Brazil. Almost all of them are public institutions, and all of them received both low- and high-risk pregnant women. Those institutions were invited to participate in the current study during a network's meeting held in Campinas, Brazil, in April 2009. Initially 26 centers accepted to participate; however 20 selected institutions were able to fully take part in the study.

### 2.3. Selection of the Electronic Research System

**Figure 1:**
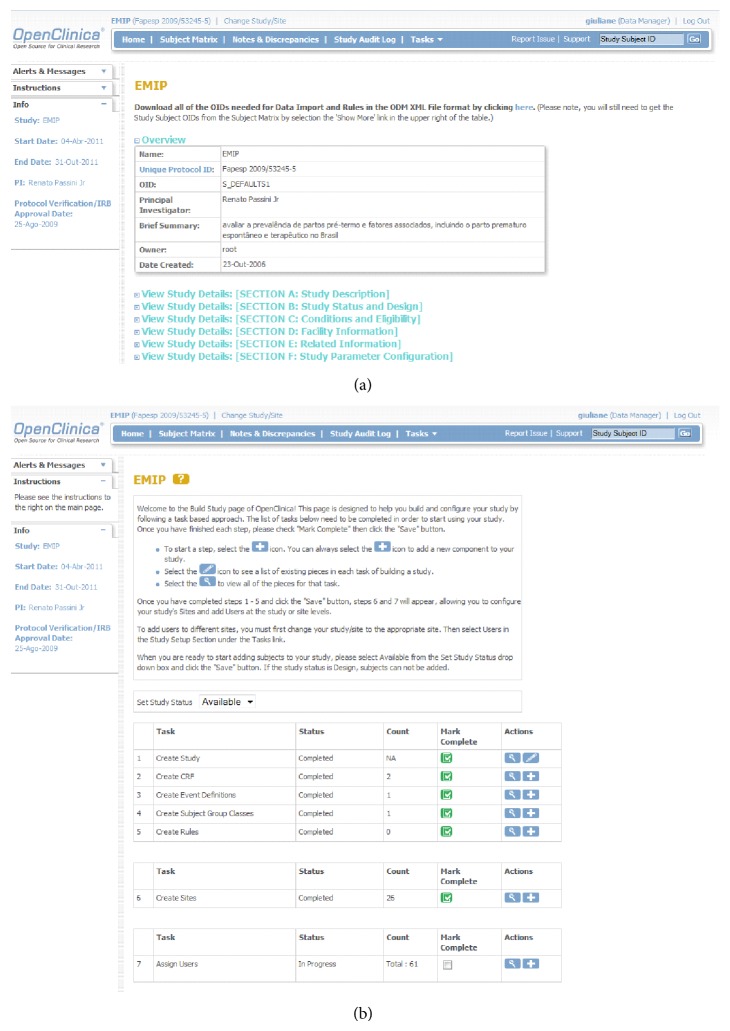
Sample screens from the online data entry and management system: (a) Coversheet for the study in* OpenClinica*. (b) Build study page of EMIP at* OpenClinica*.

The successful experience of the Brazilian Network for Surveillance of Severe Maternal Morbidity [23] using* OpenClinica* allowed us to select the same software. This internet-based system consists of an electronic platform for data entry and management of data, which is designed to support all types of clinical studies in a variety of locations [24]. We choose the free version of the system that allows forms creation, analysis and data storage, and stratification of the right of access to be granted to users working in the same study (Figure 1).

### 2.4. Data Quality

Several procedures were adopted to ensure high quality data and reliable information, including preparatory meetings, use of a detailed manual of operation, site visits, technical visits to participating centers, close monitoring of data collection and data entry, concurrent query management, inconsistency checks, and correction of database. In addition, the web-based data management system used in this study was compliant with good clinical practice (GCP) and regulatory guidelines [25], allowing differentiated user roles and privileges, password and user authentication security, electronic signatures, SSL encryption, and deidentification of protected health information (PHI). Auditing to record and monitor access and data changes aligned with a set of validation and crosschecking rules were implemented as part of the online data-management. Through this comprehensive package of data quality procedures reliable and high quality data were obtained.

### 2.5. Ethics Statement

This project has been reviewed and approved by the National Committee for Ethics in Research (CONEP, Brazilian Ministry of Health) and by the Institutional Review Board (IRB) of each site. An individual informed consent form was designed and each subject was included only after understanding and accepting the study conditions and signing it. All principles ruling research in human beings established by the Brazilian National Health Council Resolution 196/96 were followed [26]. The confidentiality of women's data and medical care was ensured regardless of whether they participated in the study or not.

## 3. Results

### 3.1. Development of Material

**Figure 2:**
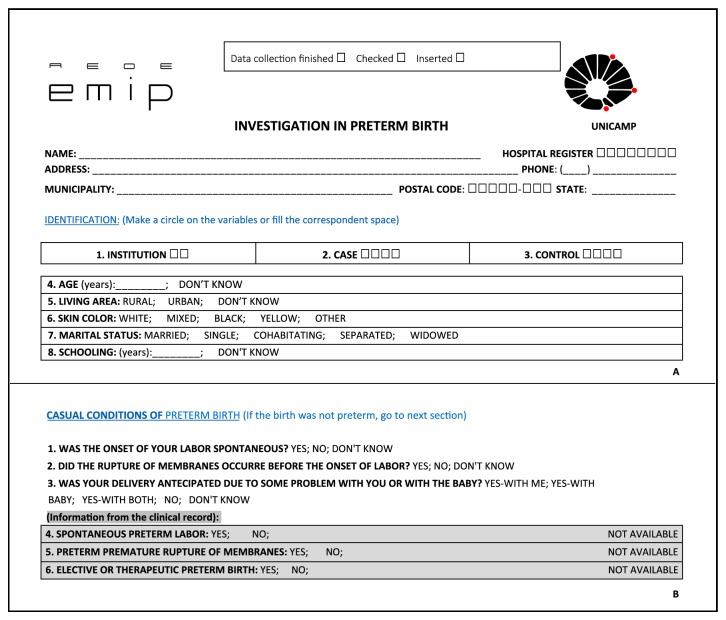
Design of questionnaire applied for data collection: (A) Checking procedures at the top of questionnaire (data collection finished, checked, and inserted) and examples of identification data (name, address, institution, case/control, age, ethnicity/skin color, marital status, literacy, etc.). (B) Information on causal conditions for preterm birth (spontaneous, premature rupture of membranes, or therapeutic). The same information in grey shadowed boxes should be extracted from the clinical records.

The research team of the coordinating center made several meetings in order to choose all variables to be included in the questionnaire, to develop a manual data collection form called “Questionnaire” that would contemplate these 306 variables. Questionnaire was organized in such a way that information from interview to postpartum women was identified differently than medical records, prenatal chart, and newborn medical records (Figure 2). As there were four different ways of collecting data in different moments, three fields at the top of questionnaire were designed—“data collection finished,” “data checked,” and “data entered”—to be completed when each step was concluded (Figure 2). The checking of data was done by local coordinator and it was a mechanism to organize and to optimize data collection.

A manual of operation was designed to provide a well-structured material that could be easily and rapidly accessed. It contained the main concepts of the study, information on the participating centers and investigators, and details of variables in order to have homogeneity and quality of information. A pretest of this questionnaire with a small sample of postpartum women in two different institutions was conducted to test its performance, and then it was finally approved. After the first meeting with all the centers, investigators were informed and trained about each variable to be studied. In this event, some suggestions were incorporated into the questionnaire and final version was performed and updated in the system as a new CRF (case report form).

### 3.2. Development of Specific Software and Hardware Tools

**Figure 3:**
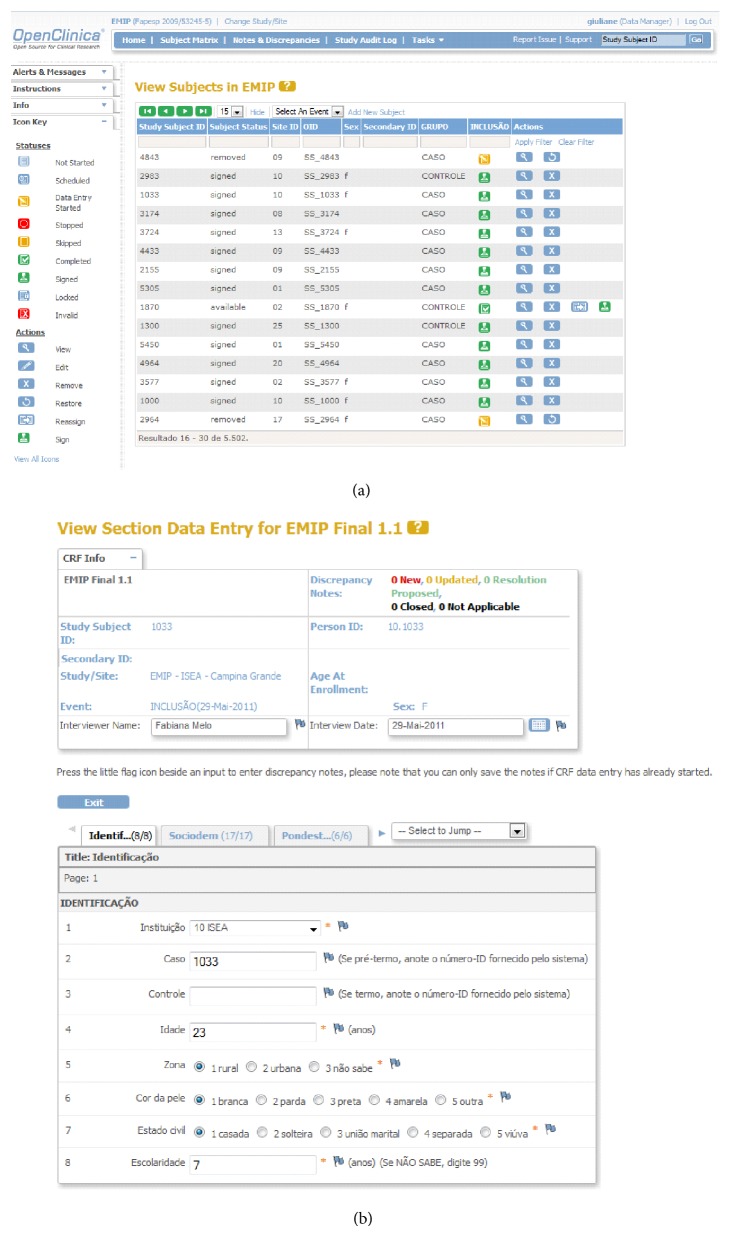
Sample screens from the online data entry and management system: (a) Form for the management of all subjects. (b) Form for data entry—13 sections with the corresponding variables.

Following selection of* OpenClinica* as the electronic data entry system for the network and registration of the study, an internet server was created in the host institution to safely store the data. The electronic address of the server was hosted in the institution's homepage with an individual safety certificate, that allowed encrypted data to be sent to the central database (Figure 1). The electronic data collection form (CRF) was developed in accordance with the standardized pattern offered by the system, with the inclusion of 13 different sections (Identification, Sociodemographic conditions, Measurements of weight and height, Obstetric history, Chronic diseases, Current pregnancy, Multiple pregnancy, Causal conditions of prematurity, Childbirth conditions, Newborn information, Spontaneous preterm birth, Preterm prelabor rupture of membranes, and Therapeutic preterm birth), containing all the variables pertinent to the study (Figure 3). Several versions had to be created and internally evaluated before the final version was reached.

A manual of operations to use* OpenClinica* database was specifically prepared for this study and a detailed training was then carried out for the development of an electronic environment to serve the network. For this purpose, usernames and passwords were created for all the research team, allowing individual access to their respective centers. Different levels of accessibility and the correspondent privileges for the inclusion and evaluation of data were granted for investigators, coordinators, supervisors, and data managers at central and local levels.

### 3.3. Implementation Process

Initially, 24 institutions accepted the invitation to participate in the study. Then, two others showed interest and were also included in this national network. All centers were coordinated by the Department of Obstetrics and Gynecology from the University of Campinas, Brazil. However, for several specific internal reasons, during the study six centers decided they were not able to continue and then were excluded, and therefore 20 selected centers from three Brazilian regions remained, 7 from the Northeast region, 11 from the Southeast region, and 2 from the South region. The characteristics of the participating centers are shown in Table 1. Each one got approval from its local IRB.

The first general study meeting was then held to introduce the study to all participants, deliver the tools, and provide a practical training. The meeting took two days and was attended by two representatives of each participating center, whose suggestions were discussed and incorporated in the study and/or questionnaire and manual of operation by the coordinating center's research team.

Questionnaire and electronic database (*OpenClinica*) were then updated and electronically sent for final appreciation, training, and approval of the participating centers. By the end of this process, the final version of the questionnaire was printed and distributed by mail to the centers according to their mean estimated number of annual deliveries (based on the previous year).

Following approval of the study, the participating centers were provided with means to operate the system* OpenClinica* for data management. Nearly half the participant centers, all of which located in Sao Paulo State, also received personal computers and printers for activities and procedures linked with the study. All the other centers had already received the same equipment for participating in a previous study of the network.

Fifteen days before starting data collection a pilot exercise was performed in order to have the system tested by the investigators in their own work environment in each center. There were however very few suggestions in this step of study. Data collection was planned to start simultaneously in all centers, which happened in April 2011. As data collection started, the forms were filled by researchers. Data entry was concluded only upon newborn discharge from hospital, with a maximum period of 60 days after delivery or, eventually, newborn death, which occurred first. Centers were getting new forms, as the cases were included in the study.

Monthly, each center sent a form with information on the number of deliveries, preterm births, live births, and stillbirths to the coordinator center. Although all preterm births were eligible for the study, the majority of centers were not able to enroll every consecutive case of preterm birth and some potential subjects were lost, mainly due to some logistic constraints. Therefore, even considering that some cases of preterm birth were not enrolled, all of them were reported and this information could be used to estimate the prevalence of preterm birth in the study sample population. On July 2012 finally we were able to have collected complete information on 5,296 births, 4,150 of them being preterm births (1,491 due to spontaneous preterm labor, 1,191 due to a preterm prelabor rupture of membranes, and 1,468 due to a therapeutic interruption of pregnancy either for a maternal or fetal condition) and a sample of 1,146 term births to be used as controls for the case-control component.

### 3.4. Analysis of the Implementation Process

The coordinator center's team was available for clarifying all possible doubts by email or phone calls. Especially in the first month of data collection, several doubts arose. Most of them were however already covered in the manual, but for two variables, a correction in the CRF of the system was necessary. After this first month, no other changes have been required.

In addition, members of the coordinator center's team visited the other centers for monitoring the study. The objective was to identify the standards each one had established to collect and insert cases/controls in the system, their institutional support for the study, and how correctly the study procedures were being followed. Specific issues on data collection and entry were also addressed during these visits. Some cases already included in the system were randomly chosen and checked against the clinical records to check for reliability of database information. In order to ensure a systematic monitoring process, instruments to identify and register information obtained from these visits were also implemented. Then a visit report was issued and sent to the specific center in order to improve their standard procedures.

The full research team in each participating center and also in the coordinating center, including the principal investigators, local investigators, coordinators, research assistants, data managers, system analyst, statistician, network manager, and accountant, proved to be essential for study development and performance. Better ideas and solutions, as well as planning for analysis and interpretation of results, arose faster and more naturally when coming from a team thinking and working altogether.

### 3.5. Extraction, Consistency Program, and Analysis of Database

When the CRF for the electronic database was built, some internal consistencies were already programmed to advice the operator in real time during data entry, especially for numeric variables. When data collection was finished, data was extracted from* OpenClinica* and converted into SPSS [27] and a program was settled in order to detect other possible errors of data consistency. This program had almost 100 commands and the majority of inconsistencies detected were identified as typing errors.

The inconsistency list that arose was first sent to central group of the study for checking and correction was performed whenever possible. Otherwise, the remaining corrections were sent to each correspondent center. This process was performed twice and took approximately eight months, and only when no more errors were detected, the data analysis was processed. Considering the participation of twenty different health facilities in the study, they were treated as clusters and therefore an analysis of cluster effect was initially performed, showing that the great majority of all study variables had very low values of intracluster correlation coefficients (ICC), denoting the desired heterogeneity in answers among centers [28]. All other analyses were planned to report measures of effects adjusted for the cluster effect of the design.

## 4. Discussion

The development of a multicenter prospective study on preterm births in Brazil was an innovative and fundamental step in order to provide information to support health policies, implementation of clinical trials, prevention, and treatment strategies. Despite the fact that participating centers were not representing all the five regions of the country, there were an expressive number of subjects evaluated and distributed among the three more populated regions of the country with a big amount of variables collected that allow for analysis of several aspects of preterm births.

The full process was guaranteed by financial resources obtained from Brazilian funding agencies. These funds enabled the large infrastructure necessary, including computers, the internet server, software, human resources to perform the surveillance and notification of data, the entire core organization of the study, and expenses involved in traveling for training meetings and technical visits.

Meetings were absolutely worth for the development of a homogeneous study. Investigators of many centers contributed in optimizing the questionnaire, with pertinent suggestions and the training allowed to update electronic and support material. The experience acquired in the National Network for the Surveillance of Severe Maternal Morbidity study [23] was fundamental in all steps of the present multicenter study implementation, including the selection of the electronic research system, the familiarity of the centers in all network aspects, and analysis methods.

The manual of operations incorporated most of the queries raised by the investigators prior to review. The entire data entry procedure was described in detail there, including illustrations taken from the system itself for guidance. Nevertheless, many of the investigators sought advice before consulting the manual and, during the consistency analysis, some errors could be avoided by the right interpretation of the manual. This showed that reading instructions prior to initiating surveillance is a mandatory step to ensure that the process flows as effectively as possible.

The large extension of questionnaire with 306 variables studied from four sources of information (interview, puerperal medical record, prenatal chart, and newborn medical record) was the most difficult point for adhesion and compliance of some centers as well as the supposed round the clock based surveillance for subject inclusion. Furthermore, some centers faced some administrative problems (strikes, lack of sufficient doctors and researchers, and others) that compromised the appropriate inclusion of cases and data collection. We believe that the characteristic of data collection was the fundamental point why the period of data collection had to be extended and it made it almost impossible to include all eligible preterm births as subjects during data collection period, with some losses. Even considering this as a possible limitation for the study, we believe however that these losses were approximately randomly distributed among centers and among all cases of preterm births in each center. Another possible limitation of the study is that this surveillance occurred mainly in tertiary referral obstetric facilities, where the prevalence of preterm birth is usually overestimated.

Two important steps for studying prematurity in Brazil (planning and implementation) were concluded. The major challenge by now is the development of strategic analysis for the data already collected, from social and biological risk factors of preterm birth to neonatal consequences of this event, in order to use these findings to understand important factors associated with preterm birth in Brazil. Preterm birth is a typical health condition involving multiple determining factors, each of them relatively well studied and explored. However, it seems that until now there was not a global real effort to put all knowledge together with the intention to enable a better full approach for women at higher risk or having preterm birth. Hopefully the information provided by this study on risk factors and other management details could be used to build a full package for screening the higher risk women, for providing preventive measures when possible, and for adequately managing women and babies in situation of preterm birth. This could theoretically be useful not only for Brazil but also for other settings, probably from other middle-income countries, sharing similar conditions. Finally, this study had a component to evaluate clinical management among participating obstetric referral centers on the three main conditions of preterm birth—spontaneous preterm labor, premature rupture of membranes, and therapeutic or provider-initiated preterm birth. We believe that the careful analysis of these data will be helpful in promoting strategies on preterm birth prevention and treatment protocols for Brazilian population in order to minimize physical and emotional consequences for children and their families.

## 5. Conclusions

The establishment of the Brazilian Multicenter Study of Preterm Birth was very much facilitated by the experience gained in some previous studies in the area in recent years, developed as part of the Brazilian Network for Studies on Reproductive and Perinatal Health. Its implementation represented the first step of a planned comprehensive assessment of preterm birth in Brazil, with detailed information that will lead to several analyses and further studies, bringing the knowledge to improve screening, diagnosis, and treatment practices in maternal and perinatal health with the final purpose of reducing the burden of this condition in the country. However, perhaps the most important goal of the current paper is to show the methodological and technical aspects involved in building and implementing such a network for studying preterm birth, especially for those dealing with the problem in low- and middle-income settings. It showed that with an organized network of personnel and health facilities interested in the topic, a relatively small budget, a well-developed research proposal, and mainly willingness, the surveillance for basic maternal and neonatal health conditions is possible and can generate a lot of important information for policy changes in public health.

## Figures and Tables

**Table 1:** Institutional characteristics of the centers participating in the study.

Characteristics	Yes	Not
Maternity ward is part of a general hospital	13	7

Has an adequate adult or maternal ICU	16	4

Woman needing intensive care should be referred to another hospital	4	16

Maternal deaths rarely occur before admission to an ICU	14	6

The access to an intensive care represents a big problem for women with severe complications	4	16

Has adequate neonatal ICU	19	1

Neonate needing intensive care should be referred to another hospital	—	20

The access to an intensive care represents a big problem for neonates with severe complications	2	18

It is the ONLY maternity providing care for high risk pregnancies in the city	8	12

It is the MAIN maternity providing care for high risk pregnancies in the city	14	6

Has a program of medical residency in Obstetrics and Gynecology	17	3

Has a program of medical residency in Pediatrics	16	4

Has a program of medical residency or training in Neonatology	16	4

Has a program of medical residency or training in Fetal Medicine	9	11

It is a secondary hospital/facility	3	

It is a tertiary hospital/facility	11	

It is a hospital/facility of higher level of complexity	6	

It is considered a referral center for care of fetuses with malformations	13	7
